# Estimation for Better Inference in Neuroscience

**DOI:** 10.1523/ENEURO.0205-19.2019

**Published:** 2019-08-01

**Authors:** Robert J. Calin-Jageman, Geoff Cumming

**Affiliations:** 1 Dominican University, River Forest, Illinois 60305; 2 La Trobe University, Melbourne, Victoria 3086, Australia

**Keywords:** estimation, neuroscience methods, statistical inference

## Abstract

The estimation approach to inference emphasizes reporting effect sizes with expressions of uncertainty (interval estimates). In this perspective we explain the estimation approach and describe how it can help nudge neuroscientists toward a more productive research cycle by fostering better planning, more thoughtful interpretation, and more balanced evaluation of evidence.

## Significance Statement

The estimation approach to inference emphasizes reporting effect sizes with expressions of uncertainty (interval estimates). The estimation approach can serve as an adjuvant towards better inference: it pushes back against over-confident claims from inadequate samples, improves comparisons of results across contexts, normalizes the publication of negligible effects, and provides a straightforward approach for planning informative studies.

## Estimation for better inference in neuroscience

Inference is at the heart of the scientific method: we collect finite datasets and then try to make reasonable generalizations about how the world works. Today *eNeuro* announces new author guidelines for statistical inference, enjoining the use of *estimation* along with or in place of *hypothesis testing*. Specifically, the guidelines ask authors to:

- Pose quantitative research questions and report quantitative answers (effect sizes).
- Countenance uncertainty in all statistical conclusions by reporting and interpreting the potential for error (interval estimates).

In some ways, this is a very subtle change in policy. The estimation approach to inference is based on the same mathematical foundations as hypothesis testing and still enables decision-making. Moreover, adopting estimation does not limit analysis options, as any hypothesis test (frequentist, Bayesian, bootstrap, etc.) can be re-expressed in terms of estimation. Why change, then? Because current norms for statistical inference are often misguided, leading to research that is wasteful, biased, and unreliable. The estimation approach can serve as an adjuvant toward better inference: it pushes back against over-confident claims from inadequate samples, improves comparisons of results across contexts, normalizes the publication of negligible effects, and provides a straightforward approach for planning informative studies. Estimation does not cure all ills, but this new policy can serve a vital role in *eNeuro*’s forward-looking efforts to promote strong rigor and reproducibility without sacrificing scientific vitality (Bernard, 2016).

In this commentary we give an overview of the estimation approach. We then give specific examples of how it can foster better inference. We conclude with some important caveats and clarifications and a list of resources that can help researchers make the transition to the estimation approach. Parts of this commentary are adapted from our previous work advocating the estimation approach (Calin-Jageman, 2017; Calin-Jageman and Cumming, 2019).

## The estimation approach

Currently most neuroscientists approach inference through null-hypothesis significance testing. In this approach, we ask a qualitative question: *Does this drug influence learning?* The data collected is then reduced down to a test statistic and *p* value, and from these we make a qualitative conclusion: *Yes, this drug influences learning*. Results are often treated as definitive, so there can be little motivation to conduct replications: *Why test the drug again now that it has been shown to work?* Although this “one-and-done” approach is common, it is not the way null-hypothesis significance testing was meant to be used. For example, Fisher argued that “a scientific fact should be regarded as experimentally established only if a properly designed experiment rarely fails to give this level of significance” (Fisher, 1926 , p. 85).

Although the testing approach now dominates the neurosciences (Szucs and Ioannidis, 2017a), this was not always the case. Much of the most enduring and fruitful research in neuroscience was completed without recourse to *p* values (Hodgkin and Huxley, 1952; Olds and Milner, 1954; Scoville and Milner, 1957; Katz and Miledi, 1968; Bliss and Lomo, 1973; Sherrington et al., 2003). In fact, hypothesis testing is a special case of a broader and older statistical tradition: estimation. Estimation is sometimes called the “New Statistics” (Cumming, 2014), because adopting this approach would be new to the many scientists who have been trained only in the testing approach. To be clear, though, estimation is not a new approach to inference, just a different application of the same thinking underlying the testing approach.

The estimation approach involves a shift in how research questions are conceptualized and reported. Rather than a qualitative question, we pose a quantitative question: *How much does this drug influence learning?* This is answered with an *effect size*, which gives a quantitative answer to the research question, and an *interval estimate*, which helps express uncertainty: *The drug improved memory by 10% with a 95% margin of error of 9%.* The effect size expresses the magnitude of difference observed in the sample (10%). The interval estimate expresses some of the uncertainty in generalizing to the population. This can be expressed as an expected magnitude of error (*95% margin of error of 9%*) or as an interval around the effect size [*95% confidence interval (1%, 19%)*]. Because some effect sizes have asymmetric expected error, reporting an interval is usually preferred. Regardless of format, an interval estimate expresses some of the uncertainty in generalizing to the population. In this example, the interval estimate indicates a need to countenance a wide range of possible effect sizes (values ∼1% are compatible with the data and so are values ∼19%).

There are different approaches to constructing interval estimates. Every null-hypothesis test with a *p* value has a corresponding confidence interval that can be reported and interpreted (*p* values and confidence intervals both represent what are called *frequentist* approaches to statistical inference). In Bayesian statistics uncertainty can be expressed with a credible interval (Kruschke, 2013) or support interval (Wagenmakers et al., 2018b). There are also randomization-based approaches to quantifying uncertainty, such as bootstrapped intervals (Ho et al., 2018). Moreover, estimates can be constructed based on specific assumptions about population distributions (parametric) or with very minimal assumptions (nonparametric). Each approach has strengths and weaknesses; researchers should quantify uncertainty in the way that best suits their research purposes and then take care to provide interpretations properly grounded in the approach they have selected.

Although there is diversity within the estimation approach, the common theme is an emphasis on uncertainty, a key aspect of good statistical practice (Wasserstein et al., 2019). Focusing on uncertainty makes salient the tentative nature of any one study and highlights the need for direct replication. Replications can then be synthesized through meta-analysis, fostering cumulative science.

## Estimation in action

**Figure 1.**
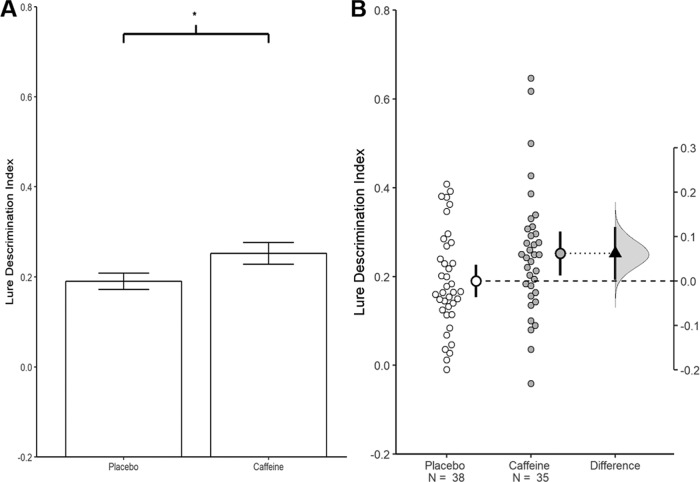
Visualizations emphasizing testing versus estimation. Both plots are from the same experiment examining the effect of caffeine on memory (Borota et al., 2014). ***A***, A traditional bar graph. The bars represent each group mean; the error bars represent the SEM. The * indicates a statistically significant difference, *p* = 0.05. ***B***, An estimation plot of the same data. In this plot the small circles represent the individual participants. The large circles with error bars represent each group mean with their 95% confidence intervals. Critically, an estimation plot emphasizes the effect size of interest for this design: the difference between the group means. This is depicted on the “difference axis” on the right. The 0 point of this axis is based on the mean of a reference group (in this case the placebo group). The filled triangle shows the difference between groups in this sample. The shaded curve shows the entire distribution of expected sampling error for the difference between the means. The error bar on the triangle indicates the 95% confidence interval for the difference between means. The confidence interval represents the range of parameter values which remain compatible with the data; that is, the variety of effect sizes that are not rejected at α = 0.05. The range of compatible values is very long and includes values that would be impossible to reliably detect with feasible sample sizes. Given this, research conclusions should be tentative and expectations for replication should be tempered. This difference plot was generated using R. The data from ***A*** and ***B*** was extracted from Borota et al. (2014).

To make the contrast between testing and estimation concrete, let us summarize the same data with both approaches (Fig. 1). Consider a recent report in *Nature Neuroscience* examining the effect of caffeine on memory (Borota et al., 2014). Participants studied images of objects and then received either 200 mg of caffeine (*n* = 35) or a placebo (*n* = 38). The next day, memory was evaluated. In the original report results were summarized using hypothesis testing, with a *t* test indicating a statistically significant enhancement in memory in the 200 mg caffeine group relative to the placebo group: *t*_(71)_ = 2.0, *p* = 0.049. From this, a qualitative conclusion was drawn: “caffeine administration enhances memory consolidation in humans” (Borota et al., 2014, p. 21). Figure 1*A* depicts how such data are often presented: a bar graph with error bars representing SEs and an asterisk to denote a statistically significant difference (in the original paper, data from each participant was also shown).

The estimation approach provides a different lens for interpreting the same data. In this approach we would ask a quantitative question: *To what extent does caffeine improve memory?* To answer this question we estimate the difference between the group means and then quantify uncertainty in this estimate due to expected sampling error: *Caffeine is estimated to improve memory relative to the placebo group by 31% with a 95% confidence interval of (0.2%, 62%)*. Figure 1*B* graphically represents this information with an estimation plot that shows the effect size in the sample and the confidence interval for the effect. Critically, the confidence interval suggests considerable uncertainty about generalizing from the sample to the world at large. If the real effect were very large (62% increase), these data would not be especially surprising. Similarly, if the real effect were vanishingly small (0.2%), these data would not be especially surprising. Moreover, this wide range of possibilities is optimistic, as it is based on uncertainty due only to sampling error with the assumption that all other sources of error and bias are negligible.

Reflecting on this uncertainty makes it clear that although this study is statistically significant the sample collected is not adequate, yielding poor signal-to-noise (the margin of error is only fractionally smaller than the observed effect). In the testing approach this is classified as *low power*. A more illuminating label is *uninformative*; the study yields too much uncertainty to provide a clear answer to the research question. The most appropriate interpretation at this point would be very modest: *caffeine probably does not impair memory consolidation*.

This striking reappraisal of the caffeine and memory study does not come from changing our epistemic standards or the statistical model used to analyze the data. The 95% confidence interval for the caffeine and memory study is just an algebraic re-expression of the *t* test used in the original paper. Specifically, the 95% confidence interval contains all the parameter values that would not be rejected using a frequentist hypothesis test at the α = 0.05 level. All values outside of the confidence interval are parameter values that are rejected at α = 0.05. The comparison between caffeine and placebo is “statistically significant” at the 0.05 level because the null hypothesis of 0 is (just barely) outside of the 95% confidence interval. Reporting the statistical test tells us that we reject this one possible parameter value. It seems clear that we can do better science by thinking critically about the parameter values that remain compatible with the data. It is this range of values that should inform our assessment of practical significance, our theory, and our planning for subsequent experiments. Thus, the new policy at *eNeuro* is to report and interpret interval estimates either in place of or alongside hypothesis tests.

In making this change, *eNeuro* joins good company at the forefront of good statistical practice. In many fields of medicine there has long been an emphasis on estimation (International Committee of Medical Journal Editors, 1997). In the behavioral sciences the American Psychological Association (2010, p. 34) enjoins researchers to “base discussion and interpretation of results on point and interval estimates” (for review, see Fidler, 2010), and interval estimates are now reported in most papers published in top psychology journals (Giofrè et al., 2017).

## Estimation thinking for an improved research cycle

Adopting estimation has several advantages (Cumming and Finch, 2001):

- Focusing on effect sizes dovetails seamlessly with the development of quantitative theories and computational models.
- Effect size estimates can be synthesized through meta-analysis, fostering cumulative science.
- Estimation is easier to understand than testing, which is really a special case of estimation (Hogben, 1957, p. 320). Teaching estimation first can help trainees better understand the uses and limits of the testing approach.
- Estimates are a natural choice for dissemination and communication with stakeholders. Modern journalistic standards specifically emphasize conveying magnitudes and uncertainty (https://www.healthnewsreview.org/).

Realizing these benefits requires more than just a rote change in how analyses are reported; it requires a different way of thinking about data that puts uncertainty at the forefront. This is critical because current norms for inference in neuroscience license problematic research practices. Neuroscience studies are often too small, yielding noisy results that are relatively uninformative ( aka, low-power research; Button et al., 2013; Dumas-Mallet et al., 2017; Szucs and Ioannidis, 2017b ). Somehow, though, this is not reflected in the public record: although noisy studies should rarely be able to detect effects, nearly all published papers report statistically significant results (Fanelli, 2012). This implausible “excess of significance” indicates that many unfavorable results have been discarded to the file drawer (Sterling, 1959; Sterling et al., 1995) or inappropriately coaxed under the threshold for statistical significance (Simmons et al., 2011; O’Boyle et al., 2017). Moreover, when statistical significance is obtained there is often insufficient attention to uncertainty, so interpretations are too confident and uninformative sample sizes are then copied forward, unwittingly perpetuating flawed research strategies. A system that is noisy and biased cannot be expected to yield reliable information, and indeed replicability seem to be low where these problems are prevalent (Boekel et al., 2015).

Here we discuss four key ways that estimation thinking can improve the neuroscience research cycle: (1) by better calibrating research conclusions to uncertainty, (2) by fostering planning and optimization toward generative lines of research, (3) by normalizing the use of inference to both rule in and rule out effects, and (4) by facilitating accurate comparison of results.

To make this discussion concrete we draw on a recent hot topic in neuroscience: the effects of intranasal oxytocin on trust and other social behaviors in humans. This topic of inquiry was initiated by a pair of prominent studies published in 2005 (Kosfeld et al., 2005; Zak et al., 2005). Since then, there has been an explosion of preclinical research and translational research on oxytocin and human social behavior. There are now grave concerns, however, about the way this research has been conducted and reported (Conlisk, 2011; Nave et al., 2015; Leng and Ludwig, 2016): studies have been too small, direct replication has been too rare, and negative results have been relegated to the file drawer. These concerns are so severe that a recent review concluded that the entire literature up to that point should be viewed with “healthy skepticism” because most published effects are probably spurious (Walum et al., 2016, p. 251). The tide now seems to be turning, with larger studies and direct replication helping to yield more clear and certain insight into how oxytocin might influence human social behavior (Liu et al., 2019). Looking back at the problems in this literature, though, brings into sharp relief fruitful avenues for improving the research cycle in neuroscience. The shortcomings we highlight are not atypical; there are several active lines of neuroscience research where selective reporting of noisy studies seems prevalent ( research on tDCS, for example; Horvath et al., 2015; Minarik et al., 2016 ; Medina and Cason, 2017).

## Estimation helps calibrate research conclusions to uncertainty

The estimation approach helps guide interpretation of data: an interval estimate that is long relative to the scale of measurement requires cautious and tentative conclusions, whereas an interval estimate that is short can warrant stronger claims. This close calibration between uncertainty and interpretation is essential: it helps match research claims to the evidence, makes clear when additional direct replication is needed, and helps set realistic expectations for subsequent research. In contrast, researchers using the testing approach often base their conclusions solely on statistical significance, treating every statistically significant result as equally and completely compelling. The *uncertainty blindness* that can occur when *p* < 0.05 licenses unequivocal claims for research that should be treated as highly tentative and generates excessive confidence in the likelihood of replication (this has been called “the replication delusion”; Gigerenzer, 2018).

**Figure 2.**
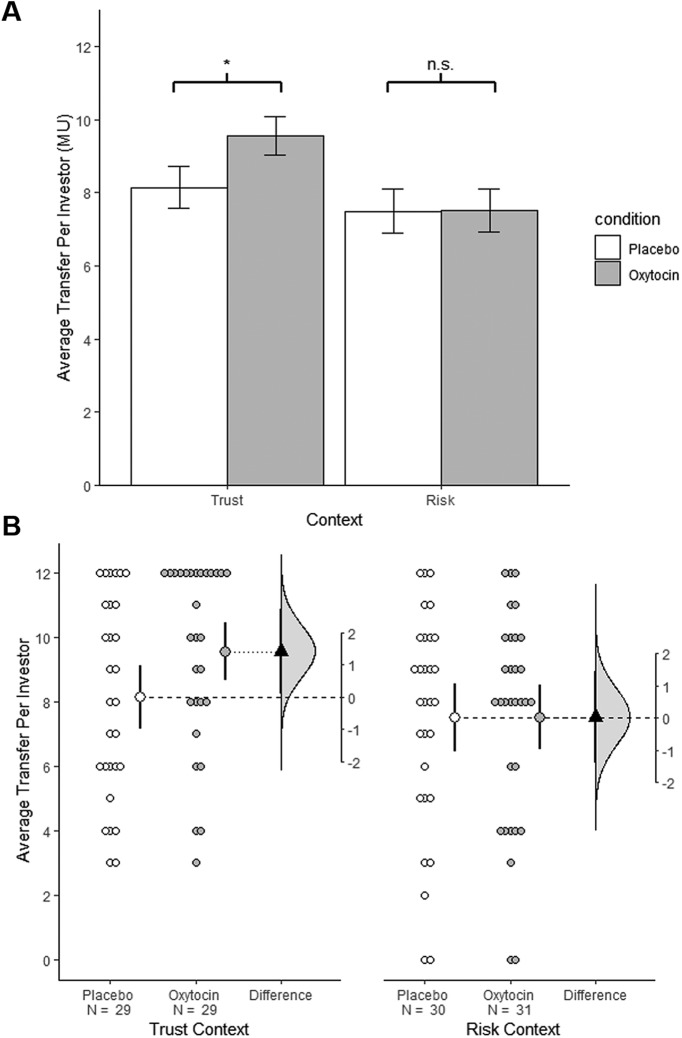
Visualizations emphasizing testing versus estimation. Both plots are from the same experiments examining the effect of oxytocin on social behavior (Kosfeld et al., 2005). ***A***, A bar graph showing the effect of intranasal oxytocin on the amount invested in a trust (left) and risk (right) game. Each bar represents group means and error bars represent ± 1 SE. There is a statistically significant effect of oxytocin in the trust game (*p* = 0.04, on tailed), but not in the risk game. The difference in statistical significance status can give the impression that oxytocin specifically influences performance in the trust game, but a formal test for an interaction is not significant (*p* = 0.23). ***B***, Estimation plots of the same data. The plots compare investment in the oxytocin and investment condition in the trust (left) and risk (right) games. Small circles represent individual participants. Large circles with error bars show group means with 90% confidence intervals (90% confidence was selected to match the stringency of the one-tailed test used in the original paper). The triangles represent the observed difference between groups, with 90% confidence intervals. The curves indicate the entire range of expected sampling error in estimating the mean difference. Note the considerable overlap in the estimated oxytocin effects in the trust and risk games. This correctly suggests that there are many compatible effect sizes in common and that these data do not support strong claims for an interaction. This figure was adapted with permission from Calin-Jageman and Cumming (2019).

As an example, consider one of the studies that helped launch research on intranasal oxytocin and human trust (Kosfeld et al., 2005). In this experiment participants received an intranasal dose of oxytocin (*n* = 29) or placebo (*n* = 29) and then played an economic trust game. There was a statistically significant effect of oxytocin (*t*_(56)_ = 1.8, *p* = 0.04, one-tailed; Fig. 2*A*, Trust context; see note at end on how these data were analyzed). In addition, there was a nonsignificant effect of oxytocin in a game involving only risk (*p* = 0.98; Fig. 2*A*, Risk context). From these results researchers made a categorical and unequivocal claim: “Oxytocin increases trust in humans” (Kosfeld et al., 2005, p. 673). Although we can all be partial to our own data, other scientists seem to have agreed with this sweeping interpretation. The study was published in *Nature* and quickly became a citation classic (>3000 citations as of January 2019, according to Google scholar). Even from the start, citations portrayed this study as unequivocal (Coan et al., 2006).

This trajectory from a single significant result to widespread acceptance of a categorical claim is the norm in our field, and yet it often represents an egregious insensitivity to uncertainty. This can be appreciated by re-expressing the statistical test used in the oxytocin study as an estimate: mean trust increased by 17.4%, 90% CI (0.5%, 34.2%; Fig. 2*B*, left; a 90% CI is used to match the stringency of the one-tailed test used by Kosfeld et al., 2005). Summarized in this way, it is clear that this study is uninformative, with a sample size too small to support a clear answer to the research question. Although the data are compatible with oxytocin producing large changes in trust, they are also compatible with oxytocin producing infinitesimal changes of no practical significance. An appropriately cautious interpretation from these data alone would be that oxytocin likely does not impair trust in humans.

Focusing on uncertainty in current results is important because it helps set realistic expectations for future studies. Many researchers erroneously believe that statistical significance means “likely to replicate” (Gigerenzer, 2018). The estimation approach helps push back against this misconception, as an interval estimate can also serve as a *prediction interval* for what to expect with a direct replication. For example, a 95% confidence interval for a mean difference will “capture” the effect size of ∼83% of same-sized replications (Cumming and Maillardet, 2006; Cumming, 2008). That is, it is expected that 83% of the time the replication effect size will be within the original 95% confidence interval. Note that the capture rate is not 95% because both studies are subject to sampling error. Moreover, this expectation is optimistic, as it assumes the original and replication studies will differ only in terms of sampling error.

Examining the interval estimate from Kosfeld et al. (2005) gives a wide range of predicted outcomes for replication studies, including effect sizes that would be practically impossible to detect. Specifically, near the lower bound of the 95% confidence interval are effect sizes that would require many thousands of participants/group to regularly detect. Consistent with this prediction, replications of Kosfeld et al. (2005) have so far obtained primarily negligible effects. A recent meta-analysis suggests the effect is small, perhaps even exactly 0 [in SD units: 0.08, 95% CI (−0.12, 0.28); Nave et al., 2015]. Whereas a focus on statistical significance might suggest this disappointing outcome is surprising (Lai et al., 2012), a focus on estimation correctly shows that this is actually an expected possible outcome, one that is within the interval estimate from the original study [in SD units, Kosfeld et al. (2005) found an oxytocin effect in the trust context of 0.49, 90% CI (0.04, 0.93)]. This is perhaps the most useful aspect of the estimation approach: it can help properly calibrate our sense of surprise across a series of results.

Strong claims from uninformative samples are not unique to the oxytocin literature. In fact, it is remarkably common for neuroscientists to unwittingly conduct and cite research that is actually uninformative ( aka under-powered; Button et al., 2013; Dumas-Mallet et al., 2017; Szucs and Ioannidis, 2017b). This means that much of what currently passes for “established” should rightly be construed as highly tentative. Reporting interval estimates helps makes more clear when a study is too uncertain to be informative. This does not have to preclude publication; sometimes an inadequate sample is unavoidable and we must make do with highly uncertain results. The key, though, is that reporting and interpreting estimates will raise needed red flags, ensuring (1) that research conclusions will be appropriately tentative, (2) that the need for direct replication with larger samples will be clear, and (3) that expectations for replications will be appropriately broad.

## Estimation fosters thoughtful research planning and optimization

Estimation is useful not only for interpreting completed studies but also for thoughtfully planning and optimizing the next study. First, estimation focuses on effect sizes and uncertainty, the inputs needed for planning samples. Second, estimation offers an intuitive approach to sample-size planning: planning for precision (Goodman, 1994; Kelley et al., 2003; Rothman and Greenland, 2018). In planning for precision (also known as the “Accuracy in Parameter Estimation” approach), researchers plan a sample size to obtain a desired level of precision (a desired margin of error). Unlike planning for power, planning for precision does not require *a priori* effect size expectations (though these are still helpful, if available). In addition, planning for precision plans to *characterize* the effect, not just to detect it, which means even if the effect is negligible the results are still informative and publishable. Planning for precision is amenable to sequential analysis (Kelley et al., 2018), so researchers can efficiently obtain a desired level of precision even in exploratory research where there is considerable uncertainty about variance in the dependent variable. The ease of planning for precision can help researchers meet their ethical obligation to avoid collecting both too little and too much data (ASA, 2016), ensuring research efforts are, to the extent possible, neither futile nor wasteful.

Forethought before initiating a study can often lead to sample-size sticker shock: obtaining an informative answer to a research question can require sample sizes that are not feasible. This is where optimization comes in: protocols can be tweaked to maximize effect sizes and minimize noise (Kraemer, 1991; MacKinnon, 2013; Meyvis and Van Osselaer, 2018). Optimization is a natural step when a laboratory’s focus is on these critical experimental outputs. In contrast, *p* values are generally too erratic (Cumming, 2008; Halsey et al., 2015) to guide optimization efforts.

Better planning is sorely needed in the neurosciences. Preclinical research seems to regularly proceed without an *a priori* sampling plan (Fritz et al., 2013; Tressoldi et al., 2013; Baker et al., 2014; Vankov et al., 2014; Tressoldi and Giofré, 2015). This neglect of best practices seems driven, in part, by a fundamental misconception that attaining statistical significance proves an adequate sample has been obtained ( Mole, 2017, 2018 ). From this flawed premise, researchers often feel comfortable setting sample sizes by following tradition or by chasing significance (Vankov et al., 2014; Goodhill, 2017). In reality, both of these approaches are problematic. Given the prevalence of uninformative research in neuroscience, relying on tradition risks copying forward the mistakes of the past *ad infinitum*. Even worse “backing into” a sample size by iteratively collecting data to obtain statistical significance provides repeated opportunities to capitalize on chance, decreasing the reliability of the results obtained (Anscombe, 1954; Simmons et al., 2011). Both of these problematic approaches to planning seem common in preclinical research, and this perpetuates the use of uninformative samples.

The oxytocin and human social behavior literature provides an acute illustration of poor planning. The study by Kosfeld et al. (2005) was one of the first to examine the effects of intranasal oxytocin on human social behavior. In this novel context it is not surprising that the sample size obtained was not well calibrated to the research question. What is surprising is that this issue was not widely recognized. Because of this, sample sizes from this exploratory work were copied forward for what should have been confirmatory work, plaguing the entire field with very poor signal-to-noise. Specifically, a recent meta-analysis found that the average published effect of oxytocin on human social behavior is fairly modest, ∼0.28 SD (Walum et al., 2016). Despite this, median sample size in this field is only 49 total participants, meaning expected sampling error is much larger (∼0.55 SD) than the typical reported effect. This is like trying to study ion channel structure with a magnifying glass: it does not mean that all the results are wrong, just that there is relatively little reason to believe them. It also means that most of these studies were launched without reasonable forethought, producing research conducted at considerable time and expense, but to little purpose.

## Estimation can mitigate publication bias

With the estimation approach, an interval can provide evidence that an effect is meaningful (the whole interval estimate is in a range of practical significance). An estimate can also provide evidence that an effect is negligible (the whole interval estimate is in a range that is not practically significant). Of course, what counts as a meaningful effect size depends on the research context and requires judgment. What is critical is that with estimation thinking both outcomes are evaluated similarly: with thoughtful attention to uncertainty and careful consideration of factors that could bias the estimate (e.g., insufficient manipulation, experimenter bias, differential dropout, procedural error, etc.).

This even-handed weighing of evidence is essential to good science, where our analytic procedures must be capable of both ruling in and ruling out effects (and of reserving judgment due to an uninformative sample). The testing approach is also suitable for these needs. In practice, though, null hypothesis testing with *p* values is often used as though it can only demonstrate effects. This is like having a neural network that can only express LTP; noise will eventually saturate the system. This one-sided approach to research is due in part to incomplete training. Current training rarely includes exposure to Bayesian techniques or equivalence testing, the *p* value approach to testing for a negligible effect (Westlake, 1972; Shuirmann, 1987; Lakens et al., 2018). Moreover, half-truths are perpetuated, with trainees strongly cautioned that nonsignificant results may be too uncertain to interpret or merely an indicator of researcher incompetency. These are half-truths because they present a false specificity: these cautions apply to all research results. That is, significant findings can also be too uncertain to interpret (uninformative), and incompetency can produce spurious effects just as easily as it can obscure real ones. These widespread misunderstandings of the testing framework help fuel publication bias, yielding a distorted published literature and a “vast graveyard of undead theories” (Ferguson and Heene, 2012).

Again, the literature on intranasal oxytocin and human social behavior provides a cautionary example. Meta-analysis shows excess significance in the published literature on this topic (Walum et al., 2016), a sure tell that many nonsignificant findings have not been published or have been massaged toward statistical significance. Indeed, one laboratory has bravely opened its file drawer for inspection (Lane et al., 2016), reporting that the five statistically significant results it has published represent just 39% of the 13 different tests the laboratory had conducted. The laboratory had tried to publish the eight nonsignificant results, but these were “rejected time and time again” (Lane et al., 2016, p. 38). Thus, the published output of this laboratory would indicate a strong effect of oxytocin, but weighing all the data the laboratory has collected indicates only a negligible effect (ibid). Again, this is not atypical; excess significance has been detected across many domains of neuroscience research (Button et al., 2013), and there is an enormous body of literature showing that nonsignificant results are far less likely to be written up, submitted, and/or published than significant results (Song et al., 2010). A research cycle that suppresses unfavorable data cannot rightly be described as scientific.

## Estimation facilitates accurate comparisons across results

Another important role of inference is in making comparisons to other contexts, conditions, or studies; this is the analysis of interactions. Within the testing approach, neuroscientists often fail to conduct a formal test for an interaction but instead rely on comparing statistical significance levels (Nieuwenhuis et al., 2011). This is invalid and frequently leads to spurious conclusions (Gelman and Stern, 2006). Two results can have the same effect size but differ in significance (e.g., due to different sample sizes). In addition, two results can be statistically significant and yet differ radically in effect size. The estimation approach helps researchers avoid this inferential trap and fosters accurate comparisons across sets of results.

For example, in the seminal experiment on intranasal oxytocin and trust (Kosfeld et al., 2005), researchers examined the effect of oxytocin on a game involving trust and on a control game involving only risk (Fig. 2*A*). Whereas oxytocin had a statistically significant effect on money transfer in the trust game (*p* = 0.04, one-tailed) it did not have a statistically significant effect in the no-trust game (*p* = 0.98). This suggests a possible interaction between oxytocin and trust, but this was not formally tested. Instead, the researchers relied on the difference in significance status to conclude that “oxytocin specifically affects trust in interpersonal interactions” (Kosfeld et al., 2005, p. 674). This is an invalid conclusion; formally testing for the interaction gives a nonsignificant result: *p* = 0.23. Within the testing framework, these data do not provide clear support of a claim for specificity.

When expressed only in terms of statistical significance, the analytic error in Kosfeld et al. (2005) is difficult to detect; it seems to have gone unnoticed by the researchers, reviewers, and numerous readers. In contrast, summarizing results with estimation makes it easier to accurately compare sets of results. For the trust experiment, oxytocin increased mean investment by $1.41, 90% CI [($0.04, $2.78); Fig. 2*B*, left]. In the non-trust experiment, oxytocin produced effectively no increase in mean investment: $0.01, 90%CI [(−$1.32, $1.35); Fig. 2*B*, right]. Although in the sample these are markedly different outcomes, there is substantial overlap in the interval estimates. This makes it clear “by eye” that there are many compatible effect sizes in common (Cumming and Finch, 2005), giving an intuitive sense that evidence for specificity is weak. To formally test for the interaction, we estimate the “difference in the difference”; the difference between each simple effect. Specifically, the oxytocin effect during the trust game ($1.41 increase) is compared with the oxytocin effect during the non-trust game ($0.01 increase), providing an estimated interaction of $1.40 90% CI (−$.52, $3.31). That is, in the sample a trust context strongly enhanced the oxytocin effect, but the data are also compatible with no interaction and even with a moderate enhancement in the non-trust context.

This analysis of the “difference in the difference” is just a quantitative way of expressing the interaction term in a 2 × 2 ANOVA. Arguably, though, the estimation approach is more transparent and easier to interpret. This clarity would be especially useful for neuroscience. Estimates of differences in results (interactions) have higher expected sampling error than estimates of simple effects, meaning that they are more likely to be uninformative (underpowered). Thus, estimation can help encourage formally correct comparisons across results in a way that is intuitive and sensitive to uncertainty.

## Some important clarifications and caveats

Estimation can help improve every aspect of the neuroscience research cycle: helping us more carefully plan, more thoughtfully interpret, more accurately compare, and more completely report neuroscience research.

- This new policy does not ban hypothesis testing; these may be reported alongside interval estimates. The use of test procedures that allow results to be judged negligible as well as meaningful are preferred (e.g., Bayesian approaches and equivalence tests; Westlake, 1972; Shuirmann, 1987; Lakens et al., 2018 ). Note that in most cases reporting an interval estimate already provides the information that would be conveyed in a hypothesis test.
- Estimates should not be used as a surrogate for hypothesis testing (e.g., mindlessly checking whether the null value is contained inside or outside the interval estimate; Fidler et al., 2004). Authors should thoughtfully evaluate interval estimates and carefully calibrate research conclusions with respect to uncertainty. Where uncertainty is high, make clear the need for replication studies with greater precision.
- Estimates must be aligned to the research question. One place to beware is with complex designs. These are often analyzed with an ANOVA and reported with a focus on a single omnibus *F* test with a form of *η*^2^ as the effect size. Omnibus tests rarely correspond to the research questions of interest, which are typically tested with a series of planned contrasts. The magnitude and uncertainty of these planned contrasts will usually be of critical interest.
- Estimation is not a panacea. It helps highlight uncertainty but it does not overcome the problems that arise with selective reporting, flexible analysis, poor model specification, etc. Moreover, statistical outputs are not the only factor in generating scientific conclusions; this also requires careful attention to the design of the study, quality of the measurement, prior knowledge, and more (McShane et al., 2019).
- Using estimation does not alter the need to clearly demarcate planned analyses from exploratory analyses. Preregistration can help make this distinction publicly verifiable.
- Selection of confidence levels (95%, 99%, etc.) should not be rote, but should be based on an evaluation of the costs/benefits of making erroneous estimates.
- There is nothing magic about the “ends” of an interval estimate; these are arbitrary relative to the selected level of confidence. Be careful not to draw sharp distinctions between values just inside an interval estimate versus those just outside: the boundaries are arbitrary and differences are matters of degree.
- Interval estimates are optimistic in that they depend crucially on statistical assumptions, which may not be perfectly realized. This provides an additional reason for not treating the boundaries of interval estimates as definitive.
- Estimation is for everyone and can be conducted within both the frequentist and Bayesian approaches to inference (Kruschke and Liddell, 2018). In this commentary, we focused on frequentist confidence intervals only because these directly re-express frequentist hypothesis tests, which are so pervasively used in the neuroscience literature.
- Just as hypothesis testing can be misunderstood and misapplied, estimation can be misunderstood and misapplied. One common misconception applies to frequentist confidence intervals. Specifically, researchers often mistakenly apply the confidence level to their specific result, claiming (erroneously) that there is a 95% probability the interval contains the true value. In fact, for frequentist confidence intervals each specific result either contains the true value or does not. The probability statement applies not to the specific result but to the procedure of interval construction. This mirrors the ambivalence of science, where we can have confidence in the process but remain anxious about each individual study. In general, researchers should strive to make sure they understand the assumptions underlying the statistical approach they have selected and to interpret their results accordingly.

## Resources for estimation

### Learning about estimation

- Kruschke (2014) provides an excellent and comprehensive introduction to Bayesian estimation; this text focuses on credible intervals. Wagenmakers et al. (2018b) provide an overview of a different Bayesian approach using support intervals.
- For those already trained in hypothesis testing with *p* values, a text by Cumming (2012) shows how to re-express standard hypothesis tests in terms of estimation. For those just starting their statistical training, Cumming and Calin-Jageman (2017) provide a textbook that teaches estimation from the outset. There are many other excellent sources on the estimation approach (Smithson, 2002; Kline, 2004).
- For online learning the Association for Psychological Science has produced a set of videos on the estimation approach: https://www.psychologicalscience.org/members/new-statistics.
- There are many excellent sources for learning about different effect size measures (Ellis, 2010; Lakens, 2013; Pek and Flora, 2018).

### Software for estimation

- ESCI is a free set of Excel modules that enables exploration of frequentist estimation concepts and planning for precision: https://thenewstatistics.com/itns/esci/.
- JASP (https://jasp-stats.org/; Marsman and Wagenmakers, 2017; Wagenmakers et al., 2018a ) and jamovi (https://www.jamovi.org/) are excellent free and open-source programs for statistical analysis. Both provide intuitive graphical-user interfaces for Bayesian and frequentist estimation for simple designs. As of version 25, SPSS has introduced Bayesian estimation for most simple designs (https://www.ibm.com/analytics/spss-statistics-software).
- There are many resources for estimation in R. One standout is the MBESS package (Kelley, 2007), which provides functions for frequentist estimation for simple and complex designs as well as routines for sample-size planning.
- The DaBest package for R (Ho et al., 2018) provides bootstrapped estimation for several designs along with outstanding visualizations. DaBest is also available in Python and as a web-application (http://www.estimationstats.com/#/).

### Planning for precision

- In the planning for precision approach researchers select a desired margin of error and then plan a sample to obtain that precision (Goodman, 1994; Rothman and Greenland, 2018).
- Planning for precision is also known as Accuracy in Parameter Estimation (AIPE). Kelley and colleagues have extensively developed concepts and tools related to this approach (Kelley et al., 2003; Maxwell et al., 2008), including approaches that allow sequential analysis to obtain a desired level of precision with maximum efficiency (Kelley et al., 2018). The MBESS package in R provides convenient functions for planning for precision (Kelley, 2007).
- Kruschke’s (2014) textbook covers the use of simulations to plan sample sizes for Bayesian estimation.
- Other notable resources include the ufs package for R (Peters and Crutzen, 2017), ESCI, and this web application from Gerben Mulder for planning experiments with multiple participants and stimuli: https://the-small-s-scientist.blogspot.com/2017/04/planning-for-precision.html.
- Another related approach worth exploring is planning for stability (Lakens and Evers, 2014).

## Notes on analysis of data from Kosfeld et al. (2005)

Kosfeld et al. (2005) compared group medians with nonparametric tests. For ease of interpretation we re-analyzed their data using parametric comparisons of means. This change in analysis strategy does not change the conclusions drawn here. Full details on how we extracted the data from Kosfeld et al. (2005) and analyzed it are contained in Calin-Jageman and Cumming (2019). In addition, the extracted data and analysis scripts are posted to https://osf.io/54n9q/.

## Synthesis

Reviewing Editor: Christophe Bernard, INSERM & Institut de Neurosciences des Systèmes Decisions are customarily a result of the Reviewing Editor and the peer reviewers coming together and discussing their recommendations until a consensus is reached. When revisions are invited, a fact-based synthesis statement explaining their decision and outlining what is needed to prepare a revision will be listed below. The following reviewer(s) agreed to reveal their identity: NONE.

Just a minor thing.

We cannot publish footnotes.

Can you include them in the main text?

thanks
